# Inter-observer and intra-observer variability in ultrasound assessment of gastric content and volume in critically ill patients receiving enteral nutrition

**DOI:** 10.1186/s13089-023-00312-x

**Published:** 2023-03-19

**Authors:** Héctor Andrés Ruiz Ávila, Carmelo José Espinosa Almanza, Cindy Yohana Fuentes Barreiro

**Affiliations:** 1 Anestesiologo-Intensivista, Unidad de Cuidados Intensivos, Hospital Universitario Nacional de Colombia, Bogotá, D.C Colombia; 2 Docente Asociado Universidad Militar Nueva Granada, Bogotá, D.C Colombia; 3 Docente Adjunto Departamento de Medicina Interna, Facultad de Medicina, Universidad Nacional de Colombia, Bogotá, Colombia; 4 Grupo de Investigación en Cuidados Intensivos de la Universidad Nacional de Colombia (GICI-UN), Bogotá, Colombia; 5 Grupo de Interés en Ultrasonido Enfocado HUN-UNAL, Bogotá, Colombia; 6 Docente Auxiliar Departamento de Medicina Interna, Facultad de Medicina, Universidad Nacional de Colombia, Bogotá, Colombia; 7 Departamento de Anestesiología, Facultad de Medicina, Universidad Nacional de Colombia, Bogotá, Colombia

**Keywords:** Stomach, Ultrasonography, Enteral nutrition, Correlation of data

## Abstract

**Background:**

The assessment of gastric content and volume using bedside ultrasound (US) has become a useful tool in emergency departments, anesthesiology departments and inpatient wards, as it provides a rapid and dynamic assessment of the gastric content of patients, which, allows making decisions regarding the risk of regurgitation or the need to adjust the strategy used to induce general anesthesia in patients with a full stomach. This assessment consists of two evaluations: a qualitative one, in which the status of the antrum, in terms of gastric content, is classified into three categories (empty, liquid content and full), and a quantitative one, where gastric volume is estimated. The objective of this study was to estimate the intra-observer and inter-observer agreement in ultrasound assessment of gastric content and volume in critically ill patients receiving enteral nutrition.

**Results:**

A total of 41 patients were included and each examiner performed 64 gastric US (*n* = 128). Participants’ average age was 56.5 years (SD ± 12.6) and 63.4% were men. Regarding the qualitative evaluation of the antrum, in supine position both examiners classified the gastric content as grade 0 in 1 gastric US (1.5%), grade 1 in 4 gastric US (6.2%) and grade 2 in 59 (92.1%). Regarding intra-observer variability in the measurement of the area of the antrum, Lin's concordance correlation coefficient (CCC), the difference of means between measurements and the 95% limits of agreement of Bland and Altman values were 0.95 (95% CI 0.940–0.977), − 0.47 cm^2^ (SD ± 1.64) and − 3.70 cm^2^ to 2.75 cm^2^, respectively, in EC1, and 0.94 (95% CI 0.922–0.973), − 0.18 cm^2^ (SD ± 2.18) and − 4.47 cm^2^ to 4.09 cm^2^ in EC2. Concerning to inter-observer variability (EC1 vs EC2) in the measurement of the area of the antrum and of gastric volume, the following CCC, mean difference between measurements and 95% limits of agreement of Bland and Altman values were obtained: measurement of the area of the antrum: 0.84 (95% CI 0.778–0.911), − 0.86 cm^2^ (SD ± 3.38) and − 7.50 cm^2^ to 5.78 cm^2^; gastric volume measurement: 0.84 (95% CI 0.782–0.913), − 12.3 mL (SD ± 49.2) and − 108.8 mL to 84.0 mL.

**Conclusions:**

The assessment of gastric content and volume using bedside US in critically ill patients on mechanical ventilation and receiving enteral nutrition showed a good intra and inter-rater reliability. Most of the patients included in the study had a high risk of pulmonary aspiration, since, according to the results of the gastric US evaluation, they had gastric volumes > 1.5 mL/kg.

## Background

In recent years, the assessment of gastric content and volume using bedside ultrasound (US) has become a useful tool in emergency departments, anesthesiology departments and inpatient wards, as it provides a rapid and dynamic assessment of the gastric content of patients, which, in turn, allows making decisions regarding the risk of regurgitation or the need to adjust the strategy used to induce general anesthesia in patients with a full stomach [1–4]. In this sense, the use of point-of-care gastric US under the I-AIM (Indication, Acquisition, Interpretation, Medical Management) strategy has allowed, among others, to determine the risk of pulmonary aspiration of gastric contents during tracheal intubation in patients with an unknown fasting status, which is very useful in terms of airway management in emergency care settings [1].

US assessment of the gastric content consists of two evaluations: a qualitative one, in which the status of the antrum, in terms of gastric content, is classified into three categories (empty, liquid content and full), and a quantitative one, where gastric volume is estimated using parameters similar to those used in methods considered to be the gold standard for this purpose, such as gastroscopy [1, 2, 5].

Due to its capacity, compared to other diagnostic methods, to determine the amount of gastric content in a fast and non-invasive way, gastric US has been proposed to be used in critically ill patients to establish their tolerance to nutritional support techniques [6]. At present, there is no gold standard for monitoring tolerance to enteral nutrition in this type of patients and current evidence does not recommend routine measurement of gastric residual volume in these patients, as it does not represent any benefit in terms of their management, and, on the contrary, it could promote an unnecessary reduction in their caloric intake [6, 7]. To be properly used in clinical practice to assess gastric content in critically ill patients, gastric US needs to be validated as a reproducible tool with low intra- and inter-observer variability. However, so far there are no studies reporting data on its diagnostic accuracy.

Considering the above, the objective of this study was to estimate the intra-observer and inter-observer agreement in ultrasound assessment of gastric content and volume in critically ill patients receiving enteral nutrition and to contribute to the validation of this useful tool in clinical decision making regarding airway management and enteral feeding in these patients.

## Methods

A correlation study was conducted at Hospital Universitario Nacional de Colombia (Bogotá, Colombia) between December 2020 and February 2021. All patients older than 18 years were admitted to the intensive care unit (ICU) on mechanical ventilation and who were receiving enteral nutrition through a nasogastric tube (at least 4 h of enteral nutrition infusion) were considered to be eligible (*N* = 198). The following patients were excluded: those who (or their legal representatives) did not agree to participate in the study; those in which, despite having agreed to participate in the study, informed consent was not obtained; pregnant women or in the postpartum period; those who, after having signed the informed consent form, died or decided (or their relatives) to change their therapeutic approach; those who had recently undergone a laparotomy, and those in which enteral nutrition was discontinued before performing the US assessment. It is worth noting that participants were recruited prospectively and that the research protocol was approved by the Ethics and Research Committee of the hospital. Assuming an alpha of 0.05, a beta power of 0.9, a mean residual gastric volume of 100 ml (SD ± 160 ml) [16], a maximum mean difference of 50 ml (SD ± 75 ml) between the observed and a maximum limit of the difference allowed between evaluations; equal to the mean of the gastric volume + 1.96 × SD of the mean difference, the calculation of the minimum sample size is estimated at 57 patients who produce 114 gastric ultrasound measurements, see table 002. The statistical software was used to calculate the sample size. MedCalc v1.19, using the estimates for the construction of a Bland and Altman plot.

**Fig. 1:**
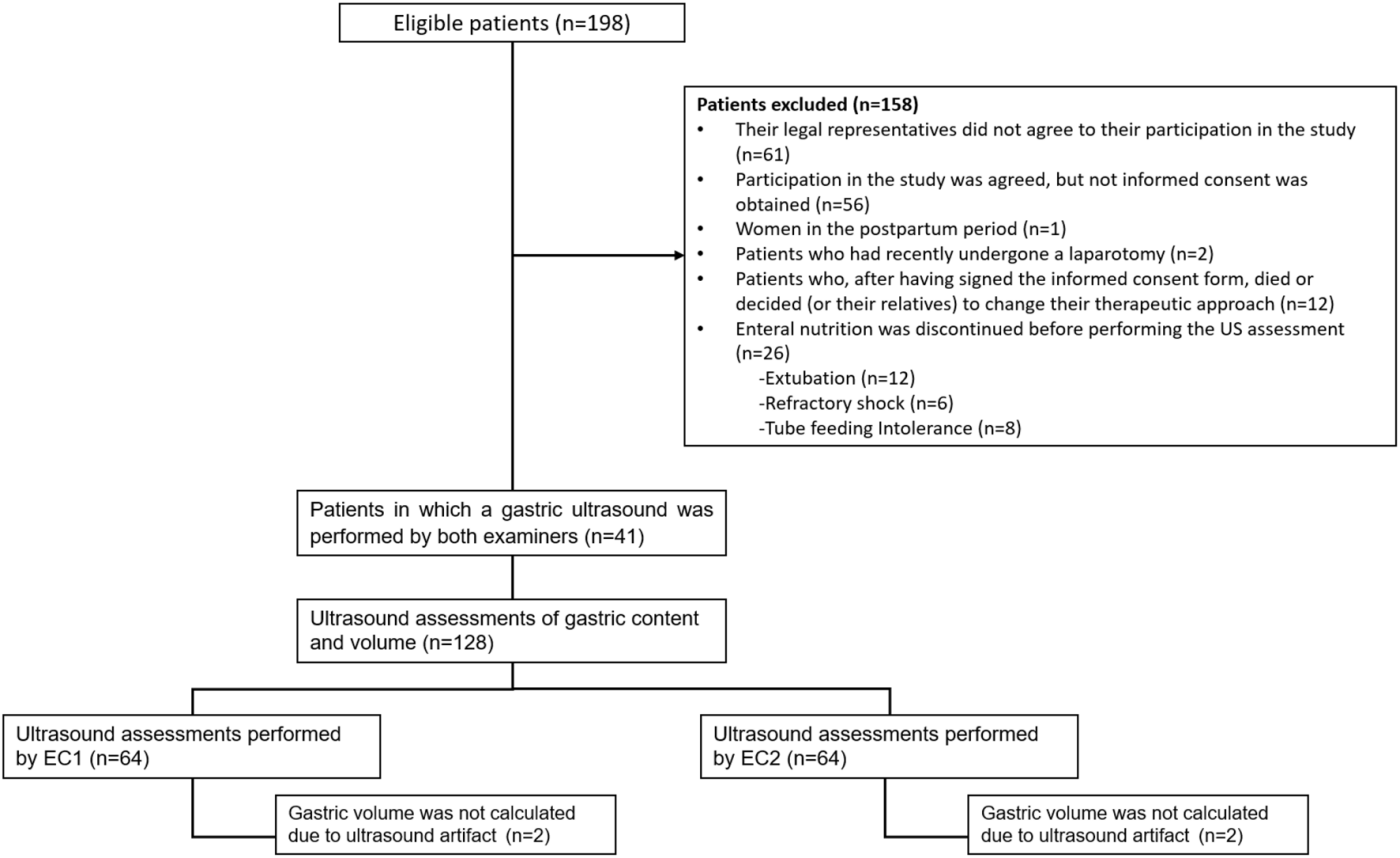
Patient selection process and number of US assessments of gastric content performed

At the ICU, one of the researchers was in charge of the daily assessment of eligible patients; then, once the informed consent form was signed by the patient or their legal representative, the equipment required for the US evaluation of their gastric content was prepared. In each patient, gastric US was carried out independently by 2 examiners who had received training in performing this procedure: EC1, a third-year anesthesiology resident (Dr. Fuentes), and EC2, an anesthesiologist specialized in intensive care (Dr. Ruiz). It is worth noting that, regarding their learning curve, each examiner had performed at least 50 gastric US. The patient selection process and the number of US assessments of gastric content are shown in Fig. 1.

**Fig. 2:**
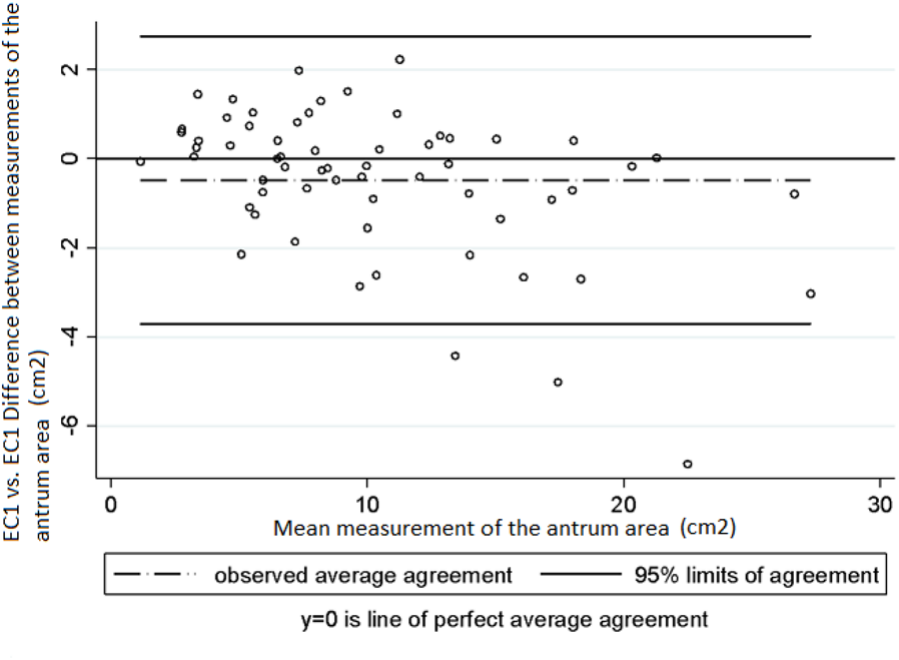
Intra-observer variability in the measurements of the area of the antrum made by EC1. Bland and Altman Analysis

**Fig. 3:**
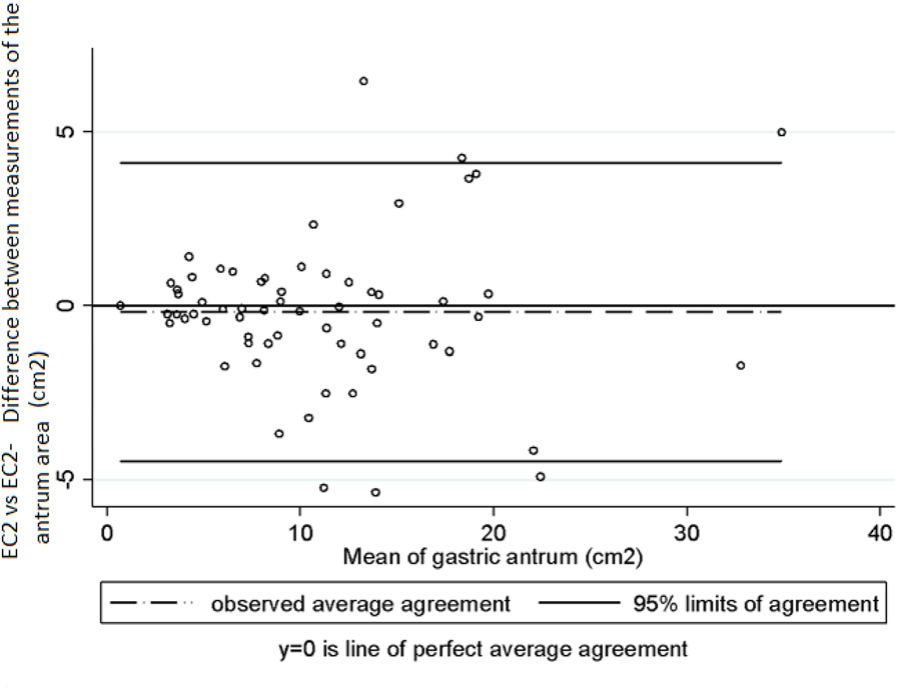
Intra-observer variability in the measurements of the area of the antrum made by EC2. Bland and Altman Analysis

**Fig. 4:**
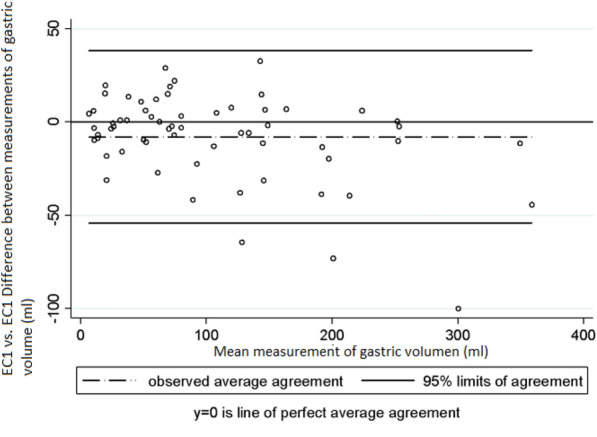
Intra-observer variability in the measurements of gastric volume made by EC1. Bland and Altman Analysis

Gastric US was initially performed with the patient in a semi-recumbent position (30–45 degrees) and then they were placed in a right lateral decubitus position, as reported in the literature [5, 7]. The 5 MHz wide band convex-array transducer was placed on the sagittal plane of the epigastric region with the index marker on the transducer pointed towards the patient’s head. Probe depth was set to 12–15 cm, using the aorta and the inferior vena cava as landmarks. Images of the antrum and the body of the stomach were obtained by tilting the transducer from right to left to achieve a general qualitative assessment of the gastric antrum, its content and the consistency of the latter (fluid or solid content) [7, 8]. The antrum was considered to be empty if it appeared flat and with juxtaposed anterior and posterior walls; to have liquid content (fluid) if there was evidence of hypoechoic content and distended walls, and to have solid content if it was distended and had a ground-glass appearance or if small specular images of intermediate echogenicity with an appearance similar to the liver parenchyma were observed [9] Figs. 2, 3, 4.

Based on the qualitative assessment, the status of the antrum was classified into three categories: grade 0: empty antrum in both supine (semi-recumbent) and right lateral decubitus positions; grade 1: fluid visible only in right lateral decubitus position (a finding suggestive of low gastric volume), and grade 2: fluid visible in the antrum in both supine and right lateral decubitus positions (a finding suggestive of greater gastric volume). Likewise, once the antrum was located, its craniocaudal and anteroposterior (AP) diameters were measured using still images obtained at rest (between peristaltic contractions) and then gastric volume was calculated using the following formula: volume (mL) = 27.0 + 14.6 × right lateral CSA (cm2)–1.28 × age (in years) [9].

Both examiners performed, independently, at least one US assessment of gastric content in each patient. In addition, if a patient continued receiving enteral nutrition for several days, both examiners could perform more gastric US, as long as the number of assessments made in the patient was the same for each examiner. To assess inter-observer variability, US measurements were blinded between EC1 and EC2 and they were performed in less than one-hour intervals. On the other hand, to assess intra-observer variability, gastric US had to be performed first with the patient in a semi-recumbent position and then with the patient in a right lateral decubitus position, and once the assessment was completed, the procedure had to be repeated exactly after one hour since the first evaluation [10, 11]. To ensure compliance with this protocol, one of the study investigators was present during the performance of all gastric US.

Regarding data analysis, the following information was obtained and recorded for all patients included in the study: clinical and sociodemographic characteristics. In addition, both examiners recorded the measurements of the diameters of the antrum in each gastric US performed and, based on this data, the following measurements were calculated: cross-sectional area of the antrum and gastric residual volume. The information was entered into a database created in the REDCap software, and data were processed and analyzed using the STATA statistical software STATA, version 14.0. The descriptive analysis of data was performed as follows: qualitative variables were summarized using absolute and relative (percentages) frequencies, and quantitative variables, using measures of central tendency and measures of dispersion. As the data showed a normal distribution (determined using histograms and box plots) medians and standard deviations were calculated [12, 13].

Intra-observer and inter-observer agreement was evaluated as follows: Cohen’s Kappa coefficient was calculated for qualitative measurements [10, 11] and, in the case of quantitative measurements, the intraclass correlation coefficient (ICC) was calculated based on the comparison of the average measurements made by both examiners (EC1 vs EC1, EC2 vs EC2 and EC1 vs EC2), which was made using the paired t-Student test or the Wilcoxon rank-sum test depending on the distribution of the data. Finally, a Bland Altman plot was constructed to establish the limits of agreement [14]. A significance level of *p* < 0.05 (two-tailed test) was considered.

## Results

A total of 41 patients were included and each examiner performed 64 gastric US (*n* = 128). Participants’ average age was 56.5 years (SD ± 12.6) and 63.4% were men. High blood pressure (36.5%), type 2 diabetes mellitus (26.8%) and cancer (4.8%) were the most frequent comorbidities; there were no cases of stage 5 chronic kidney disease or HIV infection. Since the study was conducted during the COVID-19 pandemic, respiratory failure with requirement of mechanical ventilation due to severe COVID-19 was the main cause of ICU admission (92.6%) [15]. In addition, all patients were on invasive mechanical ventilation and 39.0% were receiving vasopressors at the time gastric US was performed. The characteristics of the sample are described in Table 1.

**Table 1:** Characteristics of the sample.

Variable	Total; *n* = 41
Demographic data	
Men (%)	26 (63,41)
Age (SD ±)	56.5 (12.6)
Weight kg; (SD ±)	71.5 (15.9)
Height cm; (SD ±)	165 (10.5)
BMI kg/mt^2^ (SD ±)	25.9 (4.6)
Comorbidities	
HBP (%)	15 (36,58)
Type 2 Diabetes (%)	11 (26,82)
Cancer (%)	2 (4,87)
Therapies	
Vasopressors use (%)	16 (39,02)
Corticoids use (%)	35 (85,36)
Antibiotics use (%)	22 (53,65)
Mechanical ventilation (%)	41 (100)
Cause of admission	
Respiratory	38 (92,68)
Cardiovascular	2 (4,87)
Neurological	1 (2,43)

*BMI* Body Mass Index

Regarding the qualitative evaluation of the antrum, in supine position both examiners classified the gastric content as grade 0 in 1 gastric US (1.5%), grade 1 in 4 gastric US (6.2%) and grade 2 in 59 (92.1%), that is, there were no differences between EC1 and EC2 and their level of agreement was almost perfect (Kappa = 1.0, standard error = 0.11). On the other hand, in right lateral decubitus position, there were changes in the classification of gastric content in 3 gastric US (4.6%); this situation occurred in both examiners.

With respect to the quantitative evaluation of the antrum, the mean values and respective standard deviations (value ± SD) in supine position of the AP diameter (mm), the cross-sectional area of the antrum (mm), the area of the antrum (cm^2^) and gastric volume (mL) were 2.51 mm (SD ± 1.91), 2.25 mm (SD ± 1.16), 4.09 cm^2^ (SD ± 3.24) and 30.2 mL (SD ± 37.9) in EC1 and 2.36 mm (SD ± 1.88), 2.59 mm (SD ± 1.30), 4.29 cm^2^ (SD ± 3.11) and 30.9 mL (SD ± 36.8) in EC2. In addition, the following mean values were obtained in right lateral decubitus position: EC1 = 4.61 mm (SD ± 1.95), 2.74 mm (SD ± 0.95), 10.29 cm^2^ (SD ± 5.93) and 104.6 mL (SD ± 86.4), respectively; EC2 = 4.86 mm (SD ± 2.03), 2.75 mm (SD ± 0.99), 11.01 cm^2^ (SD ± 6.76) and 114.8 mL (SD ± 98.8). Finally, the mean gastric volume in EC1 was 101.06 ± 81.94 mL (95% CI: 80.25–121.81), while in the case of EC2 it was 113.44 ± 100.221 mL (95% CI: 87.98–138.89).

When changing from the supine position to the right lateral decubitus position, significant differences were observed between the mean measurements of the area of the antrum and gastric volume made by EC1: 4.09 cm^2^ (SD ± 3.24) vs. 10.29 cm^2^ (SD ± 5.93) (p = 0. 000) and 30.2 mL (SD ± 37.9) vs. 104.6 mL (SD ± 86.4) (*p* = 0.000), respectively; this was also the case in EC2: 4.29 cm^2^ (SD ± 3.11) vs. 11.01 cm^2^ (SD ± 6.76) (p = 0. 000) and 30.9 mL (SD ± 36.8) vs. 114.8 mL (SD ± 98.8) (*p* = 0.000). On the contrary, there were no significant differences between EC1 and EC2, both in supine and right lateral decubitus positions, in any of the four measurements considered (AP diameter, cross-sectional area of the antrum, area of the antrum and gastric volume).

Intra-observer and inter-observer variability was assessed taking into account the mean measurements of the antrum area and of gastric volume obtained in right lateral decubitus position by each examiner. Regarding intra-observer variability in the measurement of the area of the antrum, Lin’s concordance correlation coefficient (CCC), the difference of means between measurements and the 95% limits of agreement of Bland and Altman values were 0.95 (95% CI 0.940–0.977), − 0.47 cm^2^ (SD ± 1.64) and − 3.70 cm^2^ to 2.75 cm^2^, respectively, in EC1 (Graph 1), and 0.94 (95% CI 0.922–0.973), − 0.18 cm^2^ (SD ± 2.18) and − 4.47 cm^2^ to 4.09 cm^2^ in EC2 (Graph 2) [10]. In the case of gastric volume, the following values were obtained: 0.95 (95% CI 0.941–0.978), − 7.9 mL (SD ± 23.5) and–54.1 mL to 38.1 mL in EC1 (Graph 3) and 0.94 (95% CI 0.922–0.974), − 2.76 mL (SD ± 31.8) and − 65.2 mL to 59.7 mL in EC2 (Graph 4). In addition, a good intra-observer agreement was observed in both examiners in terms of US assessment of gastric content (kappa coefficient = 0.74).

**Fig. 5:**
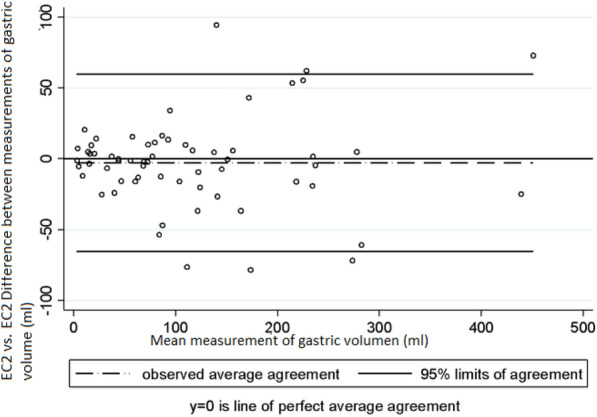
Intra-observer variability in the measurements of gastric volume made by EC2. Bland and Altman Analysis

**Fig. 6:**
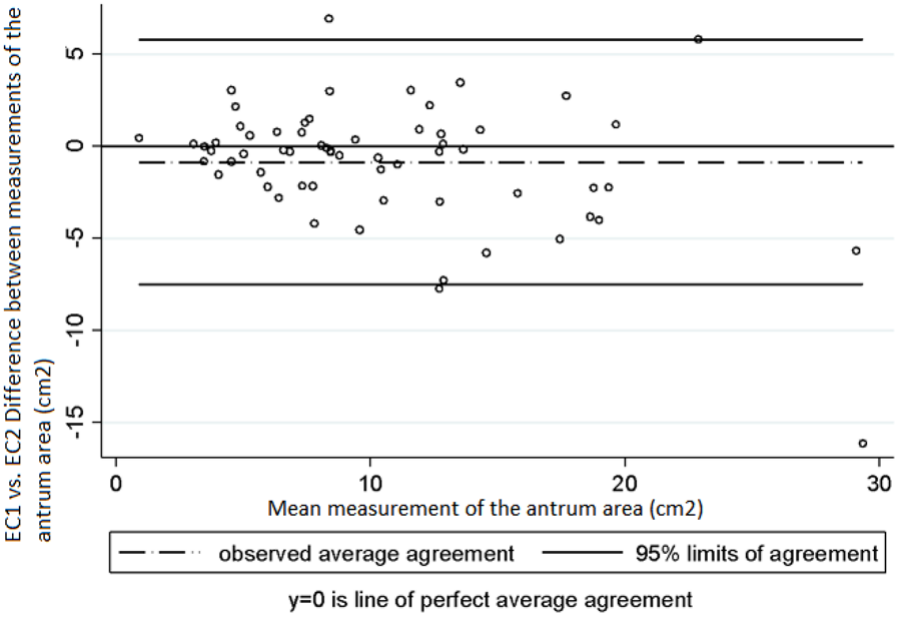
Inter-observer variability in the measurements of the area of the antrum made by EC1 and EC2. Bland and Altman Analysis

**Fig. 7:**
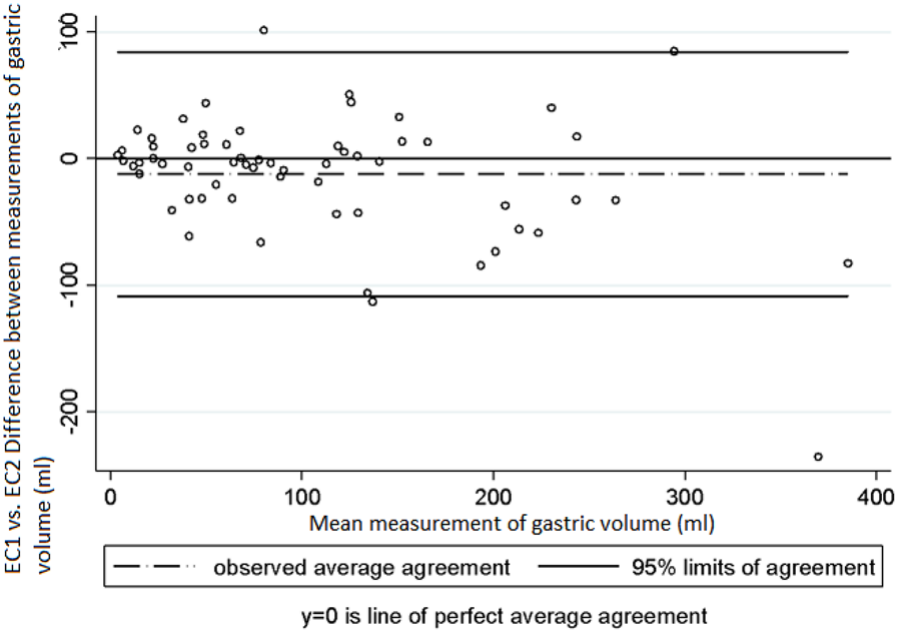
Inter-observer variability in the measurements of gastric volume made by EC1 and EC2. Bland and Altman Analysis

Regarding inter-observer variability (EC1 vs EC2) in the measurement of the area of the antrum and of gastric volume, the following CCC, mean difference between measurements and 95% limits of agreement of Bland and Altman values were obtained: measurement of the area of the antrum: 0.84 (95% CI 0.778–0.911), − 0.86 cm^2^ (SD ± 3.38) and -− 7.50 cm^2^ to 5.78 cm^2^ (Fig. 5); gastric volume measurement: 0.84 (95% CI 0.782–0.913), − 12.3 mL (SD ± 49.2) and − 108.8 mL to 84.0 mL (Fig. 6). Finally, with respect to intra-observer and inter-observer reliability in the US measurement of the cross-sectional area of the antrum, the following ICC values were obtained: ICC = 0.969 (EC1) and 0.948 (EC2) and ICC = 0.872, respectively, Fig. 7.

## Discussion

Nutritional support is fundamental in the management of critically ill patients, being enteral nutrition the nutritional support technique of choice in these patients [16, 17]. However, its use and enteral nutrition intolerance may be associated with several adverse events, such as nausea, vomiting, abdominal pain and pulmonary aspiration [18]. So far, there is no standard method to assess tolerance to this therapy in ICU patients and, in general, decisions on its use in this clinical setting are based on expert recommendations and the assessment of the clinical condition of the patient.

Bedside US offers several advantages in the management of critically ill patients. In this regard, the US assessment of gastric content strategy developed by Perlas et al. [1] and Van de Putte et al. [5], and that has also been described in multiple studies [19, 20], has become an adequate method to assess the risk of pulmonary aspiration prior to intubation in critically ill patients. In addition, although the risk of pulmonary aspiration and the US measurement of gastric content have been poorly studied in this type of patients, initial data suggest that this method has an adequate performance in the evaluation of gastric content in critically ill patients.

As mentioned above, there are only a few studies reporting data on inter-observer agreement in US assessment of gastric content. For example, Mackenzie et al. [19], in a randomized controlled clinical trial conducted in 45 healthy adult patients and in which three expert sonographers were asked to interpret 45 US and classify gastric content into three categories (presence, absence, not visible), reported that inter-observer agreement was good for the interpretation when the patient was in a right lateral decubitus position (kappa coefficient = 0.91) and moderate for the general interpretation and subxiphoid windows (kappa coefficients = 0.64 and 0.72, respectively)[19]. Similarly, Johnson et al. [21], in a randomized controlled trial in which three ultrasound experts (the one who performed the ultrasound and examiners A and B) evaluated 60 gastric US performed in healthy adult patients, found an almost perfect inter-rater agreement in the fluid content group (ICC = 0.950) and a good inter-rater reliability for the solid content and empty stomach (fasted) groups (ICC = 0.781 and 0.761, respectively) [21].

Although these studies were not specifically conducted in critically ill patients receiving enteral nutrition, their results in terms of inter-observer agreement are similar to the results of our study, where inter-rater agreement was almost perfect (kappa coefficient = 1). Furthermore, unlike the studies by Mackenzie et al. [19] and Johnson et al. [21], intra-observer agreement in the assessment of gastric content by means of US was also evaluated in the present study, obtaining a good level of agreement in both examiners (kappa coefficient = 0.74).

On the other hand, Kruisselbrink et al.[22], in a randomized controlled clinical trial carried out in Ontario in 22 healthy patients and in which three sonographers with previous experience in gastric US performed a standard US assessment of gastric volume, found an “almost perfect” intra-observer and inter-observer reliability in the ultrasound assessment of the cross-sectional area of the antrum (ICC = 0. 96 to 0.99 and 0.96, respectively); a finding similar to that reported in the present study, where intra-observer agreement (ICC = 0.969 and 0.948 for EC1 and EC2, respectively) and inter-observer agreement (ICC = 0.872) were also “almost perfect” in the US measurement of the cross-sectional area of the antrum in critically ill patients receiving enteral nutrition.

In our study, gastric content in the antrum was classified as grade 2 in 92.19% of the gastric US (*n* = 59), and gastric fluid volume was > 1.5 mL/kg in 75% of these cases. In the same vein, mean gastric volume was 101.06 ± 81.94 mL (95% CI 80.25–121.81) and 113.44 ± 100.221 mL (95% CI 87.98–138.89) in EC1 and EC2, respectively. These findings suggest that critically ill patients receiving enteral nutrition through a nasogastric tube are at high risk of pulmonary aspiration [1, 2, 5].

Additionally, the present study compared the intra-observer agreement in US assessment of gastric content of an examiner trained and certified in gastric US with the intra-observer agreement of an examiner who, despite having performed at least 50 qualitative and quantitative US assessments of gastric volume and content, had not received any formal training in gastric US. In the case of the qualitative assessment of the gastric content by means of US, intra-observer agreement was similar in both examiners; however, in the case of quantitative assessment, the mean difference in gastric volume was statistically significant (− 7.99 mL ± 23.56 mL; *p*-value = 0.009) in the examiner without training in gastric US, while in the examiner with training in gastric US this difference was not significant (− 2.77 mL ± 31.89 mL; *p*-value = 0.497). This difference in the means of gastric volume can be considered acceptable, taking into account that basal gastric secretions generate fasting gastric residual volumes of up to 1.5 mL/kg without this representing a significant risk of pulmonary aspiration (approximately 100–110 mL in the average adult population) [1, 2, 20].

Although the training specifications necessary for the performance of a reproducible gastric US assessment have not yet been defined, Arzola et al. cited by Kruisselbrink et al. [22], suggest that the sonographer should perform at least 33 gastric US under the supervision of an expert to achieve an accuracy rate of 95% in the qualitative US evaluation of gastric content. On the other hand, according to these authors, since quantitative US evaluation of gastric volume requires additional steps, it is logical to assume that the examiner must have performed a much higher number of gastric US to achieve a similar accuracy rate [22]. Considering the above, further studies are required to establish the optimal learning curve necessary for trainees to perform an adequate US assessment of gastric content.

This is one of the first studies that evaluates intra-observer and the inter-observer agreement in US assessment of gastric volume and content (being the latter classified in grades) in critically ill patients receiving enteral nutrition, which is undoubtedly its main strength, since the results reported here may contribute to the validation of this gastric content classification method in this type of patients and, therefore, its implementation in clinical practice.

On the other hand, this study has some limitations. First, it is a nonrandomized observational single-center study; however, its sample size is appropriate for the assessment of intra-observer and inter-observer agreement. Second, the COVID-19 pandemic had a great impact on the study population (critically ill patients on mechanical ventilation and receiving enteral nutrition), since 95.31% of the patients who were considered eligible for inclusion were admitted to the ICU due to respiratory failure resulting from severe COVID-19. Third, during the position changes required for achieving a proper US assessment of the gastric content and volume, five patients regurgitated the enteral nutrition formula, which, besides peristaltic contractions and gastric emptying, adds an element of variability in successive gastric ultrasound measurements.

## Conclusions

The assessment of gastric content and volume using bedside US in critically ill patients on mechanical ventilation and receiving enteral nutrition showed a good intra and inter-rater reliability.

In addition, most of the patients included in the study had a high risk of pulmonary aspiration, since, according to the results of the gastric US evaluation, they had gastric volumes > 1.5 mL/kg. In this sense, further studies aimed at determining whether US assessment of gastric volume can predict the risk of intolerance to enteral nutrition in critically ill patients are required.

Finally, the findings of this study raise some questions regarding the training required to ensure a valid and reproducible US assessment of gastric content and volume.

## Data Availability

The data that support the findings of this study are available from Hospital Universitario Nacional repository (http://webapp.hun.edu.co/redcap/index.php). Restrictions apply to the availability of these data, which were used under license for this study. Data are available with the permission of Hospital Universitario Nacional, contact information: investigacion@hun.edu.co.

## References

[1] Perlas A, Van de Putte P, Van Houwe P, Chan VW (2016). I-AIM framework for point-of-care gastric ultrasound. Br J Anaesth.

[2] Tatsumi H (2019). Enteral tolerance in critically ill patients. J Intensive Care.

[3] Ingvild Holtan-Hartwig, Rise Johnsen L, Dahl V, Haidl F (2021). Preoperative gastric ultrasound in surgical patients who undergo rapid sequence induction intubation. Trends Anaesth Crit Care.

[4] El-Boghdadly K, Wojcikiewicz T, Perlas A (2019). Perioperative point-of-care gastric ultrasound. BJA Educ.

[5] Van de Putte P, Perlas A (2014). Ultrasound assessment of gastric content and volume. Br J Anaesth.

[6] Sharma V, Gudivada D, Gueret R, Bailitz J (2017). Ultrasound-assessed gastric antral area correlates with aspirated tube feed volume in enterally fed critically Ill patients. Nutr Clin Pract.

[7] McClave SA, Lukan JK, Stefater JA, Lowen CC, Looney SW, Matheson PJ (2005). Poor validity of residual volumes as a marker for risk of aspiration in critically ill patients. Crit Care Med.

[8] Hamada SR, Garcon P, Ronot M, Kerever S, Paugam-Burtz C, Mantz J (2014). Ultrasound assessment of gastric volume in critically ill patients. Intensive Care Med.

[9] Perlas A, Mitsakakis N, Liu L (2013). Validation of a mathematical model for ultrasound assessment of gastric volume by gastroscopic examination. Anesth Analg.

[10] Vach W (2005). The dependence of Cohen’s kappa on the prevalence does not matter. J Clin Epidemiol.

[11] Chang CH (2014). Cohen’s kappa for capturing discrimination. Int Health.

[12] Bhattacherjee A. Quantitative analysis: Descriptive statistics. 2019. https://usq.pressbooks.pub/socialscienceresearch/chapter/chapter-14-quantitative-analysis-descriptive-statistics/

[13] Burdenski TK (2000). Evaluating univariate, bivariate, and multivariate normality using graphical procedures.

[14] Giavarina D (2015). Understanding bland altman analysis. Biochem Med.

[15] El Coronavirus en Colombia. https://coronaviruscolombia.gov.co/Covid19/

[16] Valencia E, Marin A, EA C. (2016) Guias de soporte metabolico y nutricional - aspen.

[17] Singer P, Blaser AR, Berger MM, Alhazzani W, Calder PC, Casaer MP (2019). ESPEN guideline on clinical nutrition in the intensive care unit. Clin Nutr.

[18] Gohel TD, Kirby DF, Seres DS, Van Way I, Charles W (2016). Access and complications of enteral nutrition support for critically Ill patients. Nutrition support for the critically Ill.

[19] Mackenzie DC, Azad AM, Noble VE, Liteplo AS (2019). Test performance of point-of-care ultrasound for gastric content. Am J Emerg Med.

[20] Bouvet L, Zieleskiewicz L, Loubradou E, Alain A, Morel J, Argaud L (2020). Reliability of gastric suctioning compared with ultrasound assessment of residual gastric volume: a prospective multicentre cohort study. Anaesthesia.

[21] Johnson EJ, Morbach J, Blake C, Pecka S (2021). Sensitivity and specificity of gastric ultrasonography in determination of gastric contents. AANA J.

[22] Kruisselbrink R, Arzola C, Endersby R, Tse C, Chan V, Perlas A (2014). Intra- and interrater reliability of ultrasound assessment of gastric volume. Anesthesiology Julio De.

